# Supplementary material for: Self-directed online machine learning for topology optimization
Source: Nat Commun. 2022 Jan 19;13:388. doi: 10.1038/s41467-021-27713-7 (PMC8770634; doi:10.1038/s41467-021-27713-7)
Supplement: Supplementary file 1 — Supplementary Information [file 41467_2021_27713_MOESM1_ESM.pdf]

Supplementary Information

Self-Directed Online Machine Learning for Topology Optimization

Changyu Deng[1], Yizhou Wang[2], Can Qin[2], Yun Fu[2], and Wei Lu[1,3,*]

[1]Department of Mechanical Engineering, University of Michigan, Ann Arbor, MI 48109, United States
[2]Department of Electrical and Computer Engineering, Northeastern University, Boston, MA 02115, United States
[3]Department of Materials Science and Engineering, University of Michigan, Ann Arbor, MI 48109, United States
[*]Corresponding author: weilu@umich.edu

Table of Contents

Section 1: Supplementary table and figures 3

- Supplementary Table 1: Average wall time within a loop 3
- Supplementary Fig. 1: Objective (energy) and prediction error of the compliance minimization problem with 5×5 variables 4
- Supplementary Fig. 2: Evolution of the solution from SOLO for the compliance minimization problem with 5×5 variables 5
- Supplementary Fig. 3: Evolution of the solution from SOLO for the compliance minimization problem 11×11 variables 6
- Supplementary Fig. 4: Evolution of the solution from SOLO-G for the fluid-structure optimization problem with 20×8 mesh 7
- Supplementary Fig. 5: Repeating SOLO-G for the fluid-structure optimization problem with 20×8 mesh 7
- Supplementary Fig. 6: Repeating SOLO-R for the fluid-structure optimization problem with 20×8 mesh mesh 8
- Supplementary Fig. 7: Evolution of the solution from SOLO-G for the fluid-structure optimization problem with 40×16 mesh 9
- Supplementary Fig. 8: Perturbation of the optimum from SOLO-G for the fluid-structure optimization problem with 40×16 mesh. 10
- Supplementary Fig. 9: Repeating SOLO-G for the fluid-structure optimization problem with 40×16 mesh 11
- Supplementary Fig.10: Perturbation of the optimum from SOLO for the heat transfer enhancement problem 12

Section 2: Theory on convergence 13

- Section 2.1: Formulation and theorem 13
- Section 2.2: Preliminaries 15
- Section 2.3: Proof 16

1 Supplementary table and figures

Supplementary Table 1: Average wall time within a loop of SOLO. There are three major steps in each loop: FEM calculation to obtain corresponding objective function values, DNN training, and optimization which searches for the optimum based on DNN's prediction. We give a very rough estimate on our personal computer (CPU: Intel i7-8086K, GPU: NVidia RTX 2080 Super). *Italic numbers* indicate GPU computing and the others are computed entirely on CPU. Actual running time is sensitive to hardware environment, software packages, parameter setting and so forth. Further, FEM calculation is approximately proportional to the number of additional samples per loop; training time depends on existing training data obtained from previous loops; optimization depends on the number of function evaluations. Similar to other SMBO methods, our surrogate model introduces overhead computation. In the compliance and fluid problems, the overhead is comparable with FEM calculation time, yet it is almost negligible considering the huge benefit of reducing FEM calculations from $10^5 \sim 10^8$ (see the table) to $10^2 \sim 10^4$; besides, we chose relatively simple problems and thus each calculation only cost < 0.5 s for compliance problems and < 6 s for fluid problems; smaller portion of the overhead is expected for more complicated problems with higher FEM computation time. When the problem becomes more complicated in the heat example, a larger advantage of our method can be observed. In the three truss problems, our focus is on the reduction of FEM calculations rather than computation time since the problems are fast to calculate.

Problem	Number of additional samples	Wall time /s		
		FEM	Training	Optimization (evaluations)
Compliance 5×5	100	40	35	70 (2×10^5)
Compliance 11×11	1000	500	150	1000 (4×10^6)
Fluid 20×8 (G)	10	35	*10*	*35* (1×10^8)
Fluid 20×8 (R)	100	350	*20*	*35* (1×10^8)
Fluid 40×16 (G)	10	60	*25*	*140* (2×10^8)
Heat 10×10	200	40000	*25*	*200* (4×10^8)
Truss 72	10	*0.02*	*20*	*300* (1×10^9)
Truss 432	50	*0.05*	*150*	*500* (1×10^9)
Truss 1008	100	*0.15*	*1500*	*1500* (2×10^9)

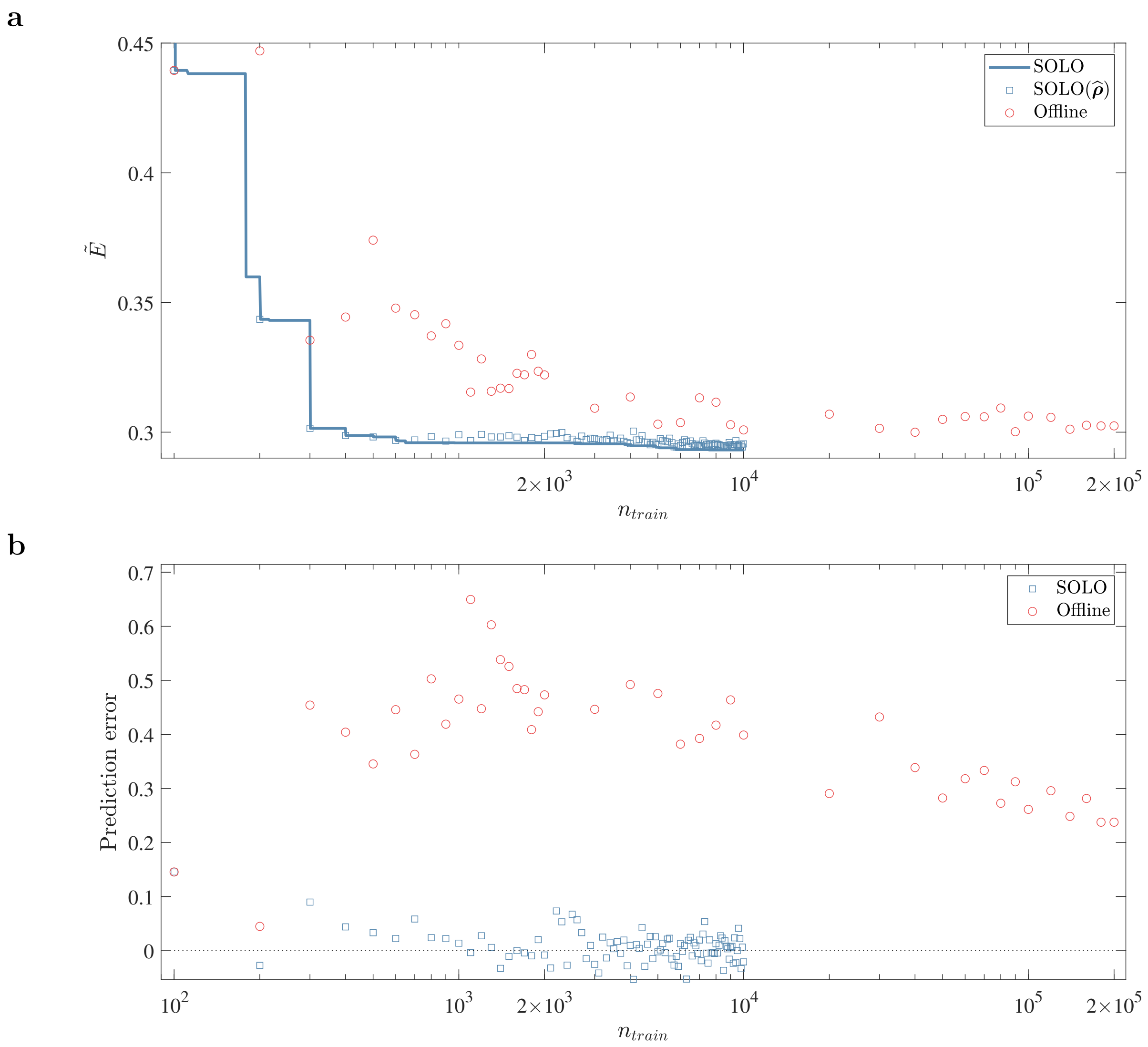

Supplementary Fig. 1: Objective (energy) and prediction error of the compliance minimization problem with 5×5 variables. a, Dimensionless energy as a function of n_{train}. For SOLO, the solid line denotes the best objective values and the squares denote $\widetilde{E}(\widehat{\boldsymbol{\rho}})$. **b**, Energy prediction error of $\widehat{\boldsymbol{\rho}}$.

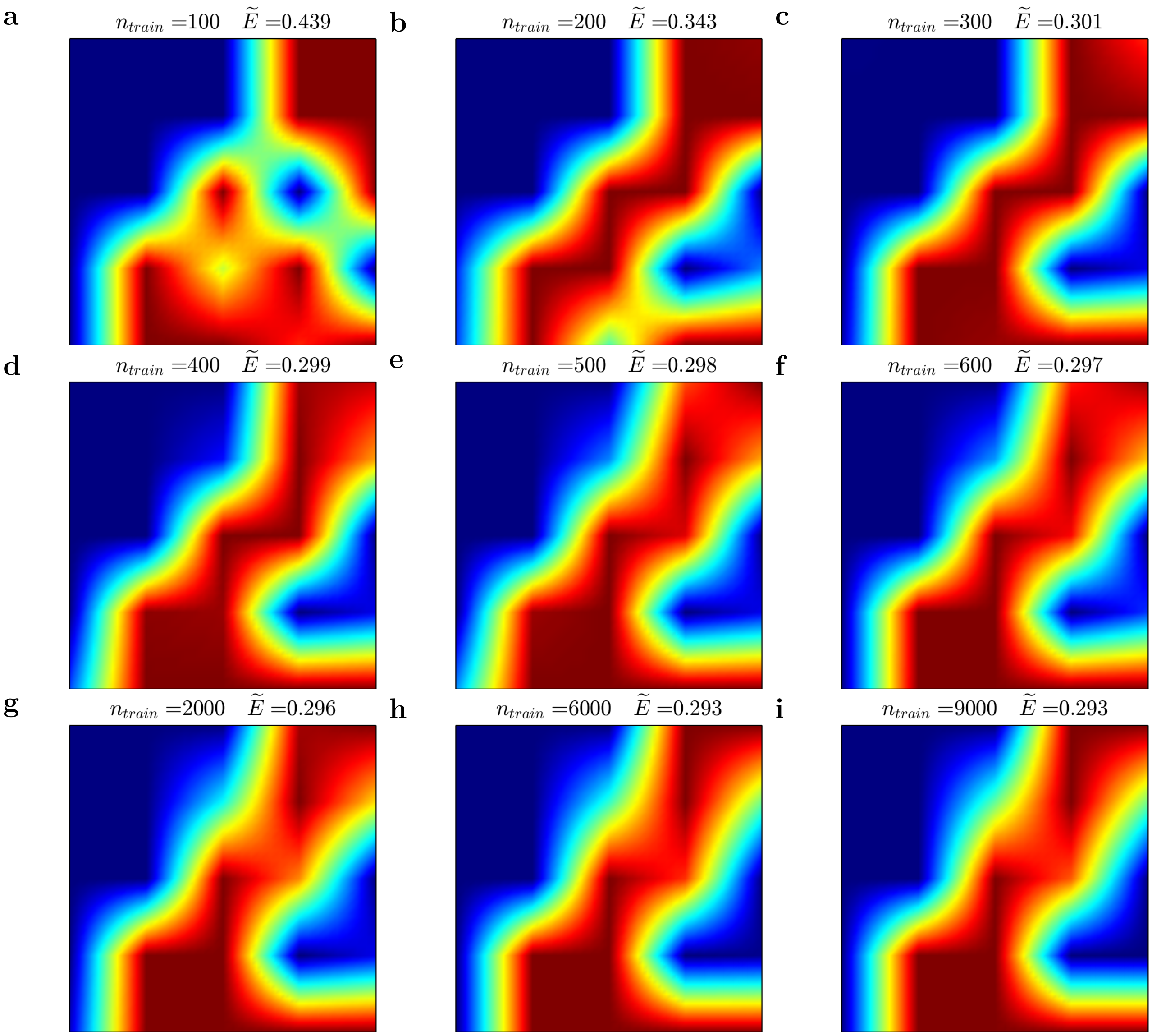

Supplementary Fig. 2: Evolution of the solution from SOLO for the compliance minimization problem with 5×5 variables. Each plot is the best among n_{train} accumulated training data and the corresponding energy $\widetilde{E}$ is marked. There is no obvious change after hundreds of training samples.

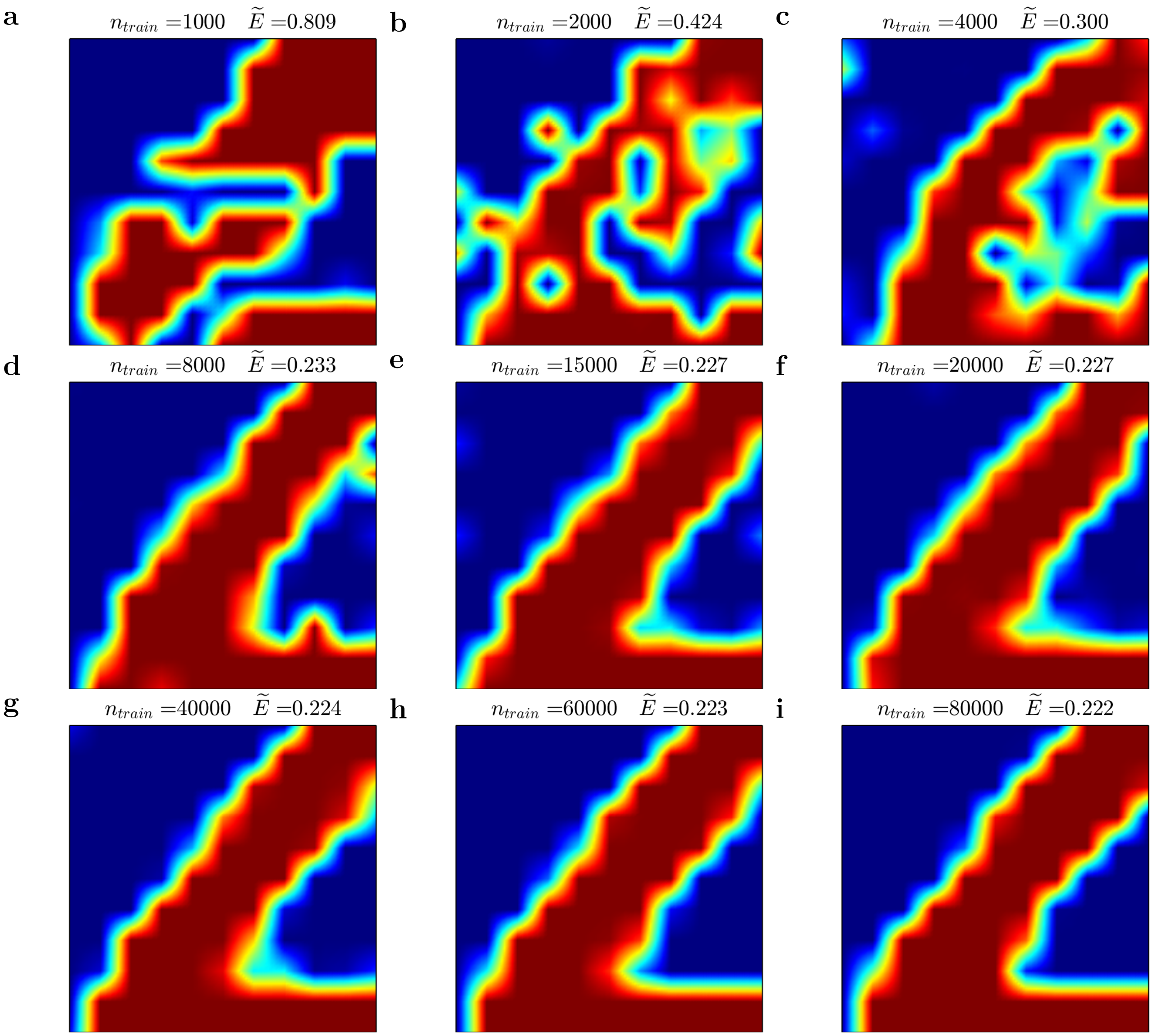

Supplementary Fig. 3: Evolution of the solution from SOLO for the compliance minimization problem 11×11 variables. Each plot is the best among n_{train} accumulated training data and the corresponding energy $\widetilde{E}$ is marked. There is no obvious change after ten thousand training samples.

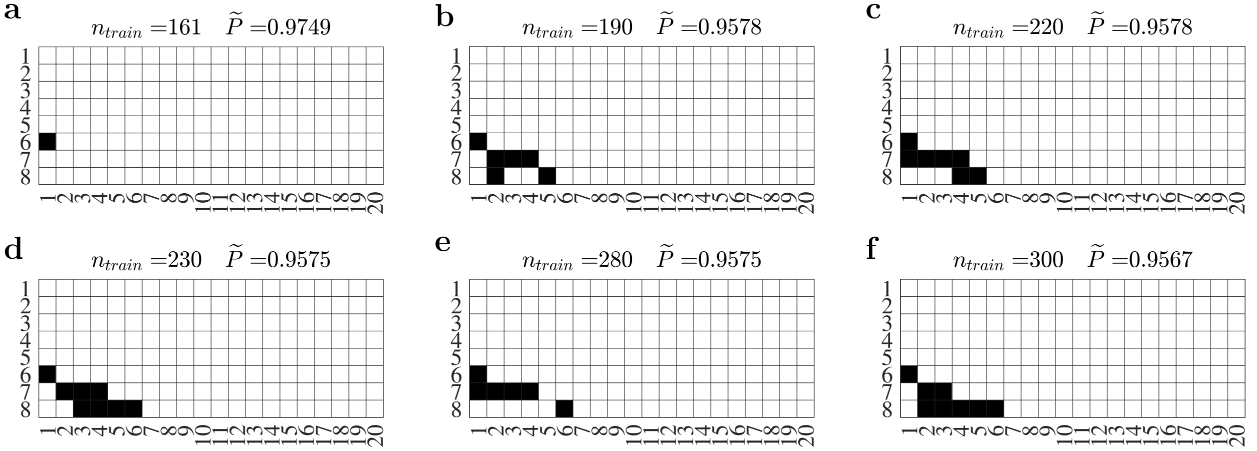

Supplementary Fig. 4: Evolution of the solution from SOLO-G for the fluid-structure optimization problem with 20×8 mesh. Each plot is the best among n_{train} samples.

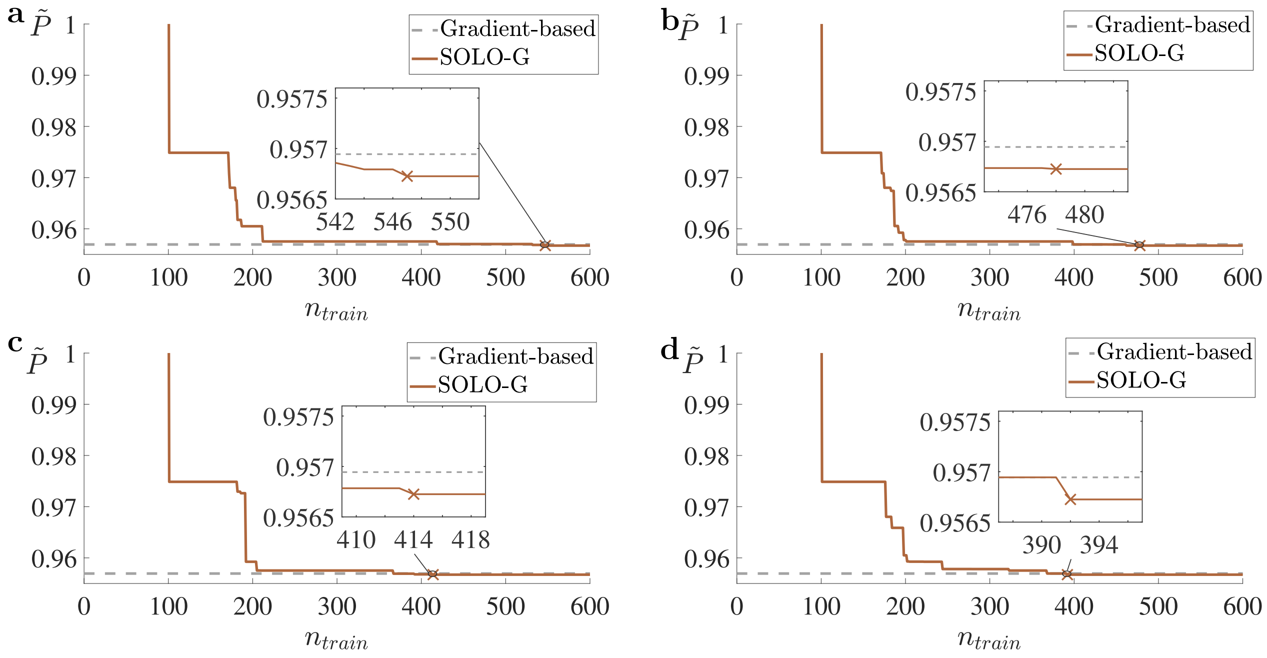

Supplementary Fig. 5: Repeating SOLO-G for the fluid-structure optimization problem with 20×8 mesh. All configurations are the same as Fig. 4b except different random seeds. They obtain the same objective $\widetilde{P}$ despite different convergence rate.

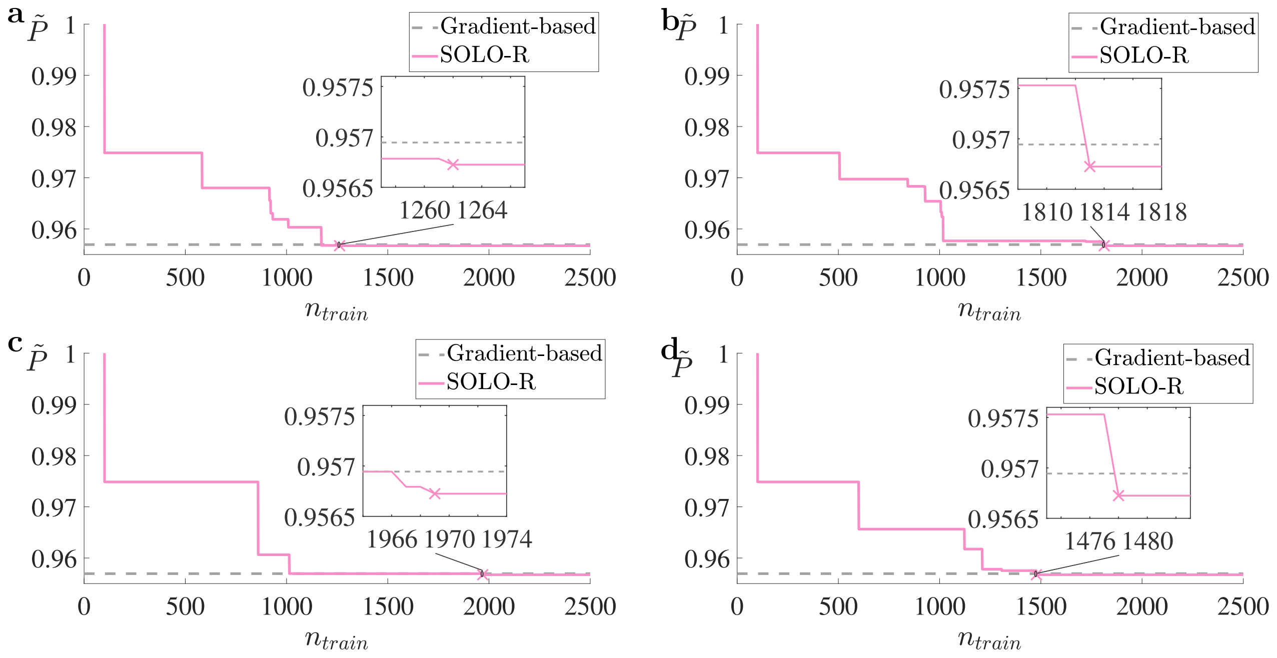

Supplementary Fig. 6: Repeating SOLO-R for the fluid-structure optimization problem with 20×8 mesh. All configurations are the same as Fig. 4b except different random seeds. They obtain the same objective $\widetilde{P}$ despite different convergence rate.

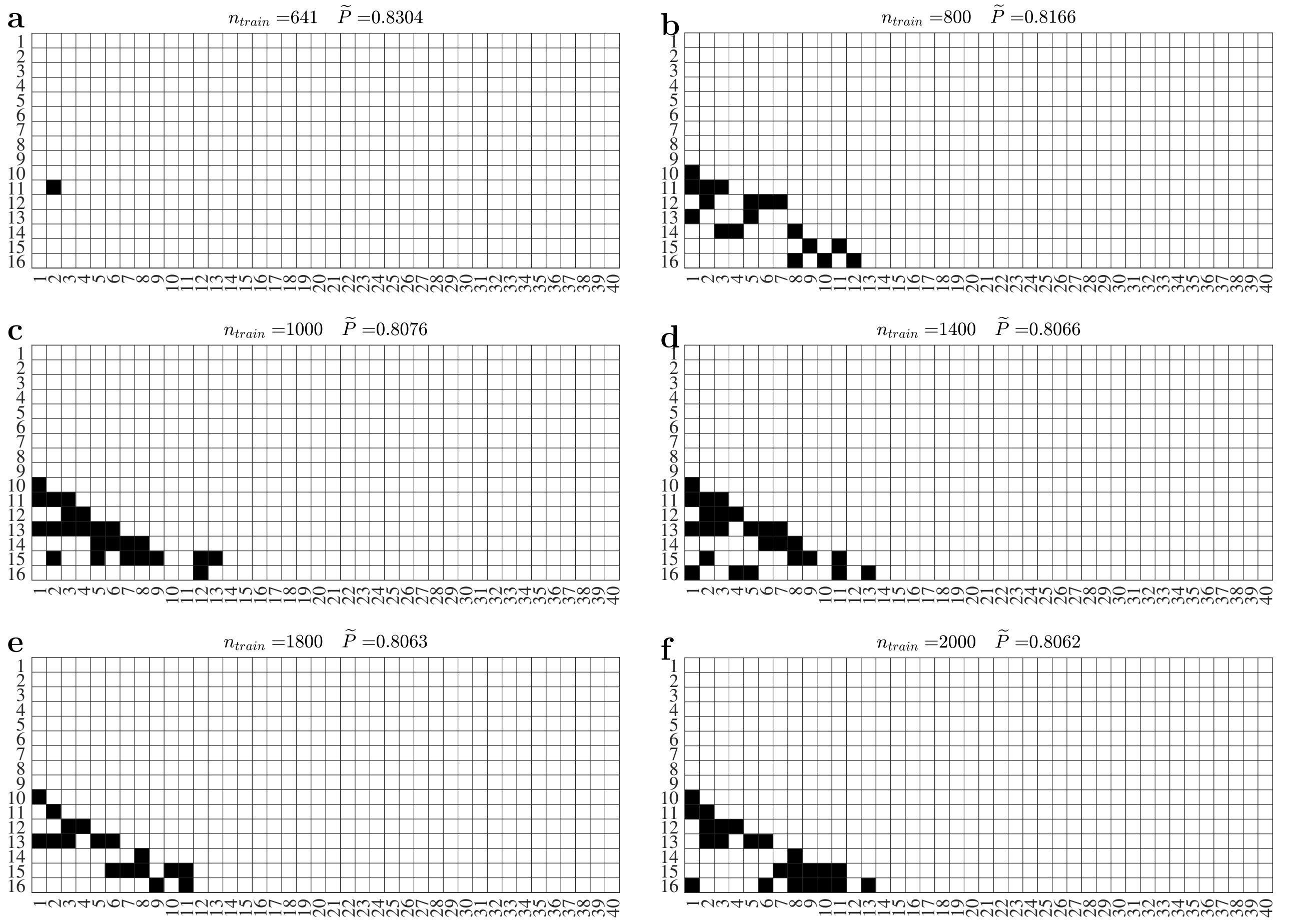

Supplementary Fig. 7: Evolution of the solution from SOLO-G for the fluid-structure optimization problem with 40×16 mesh. Each plot is the best among n_{train} samples.

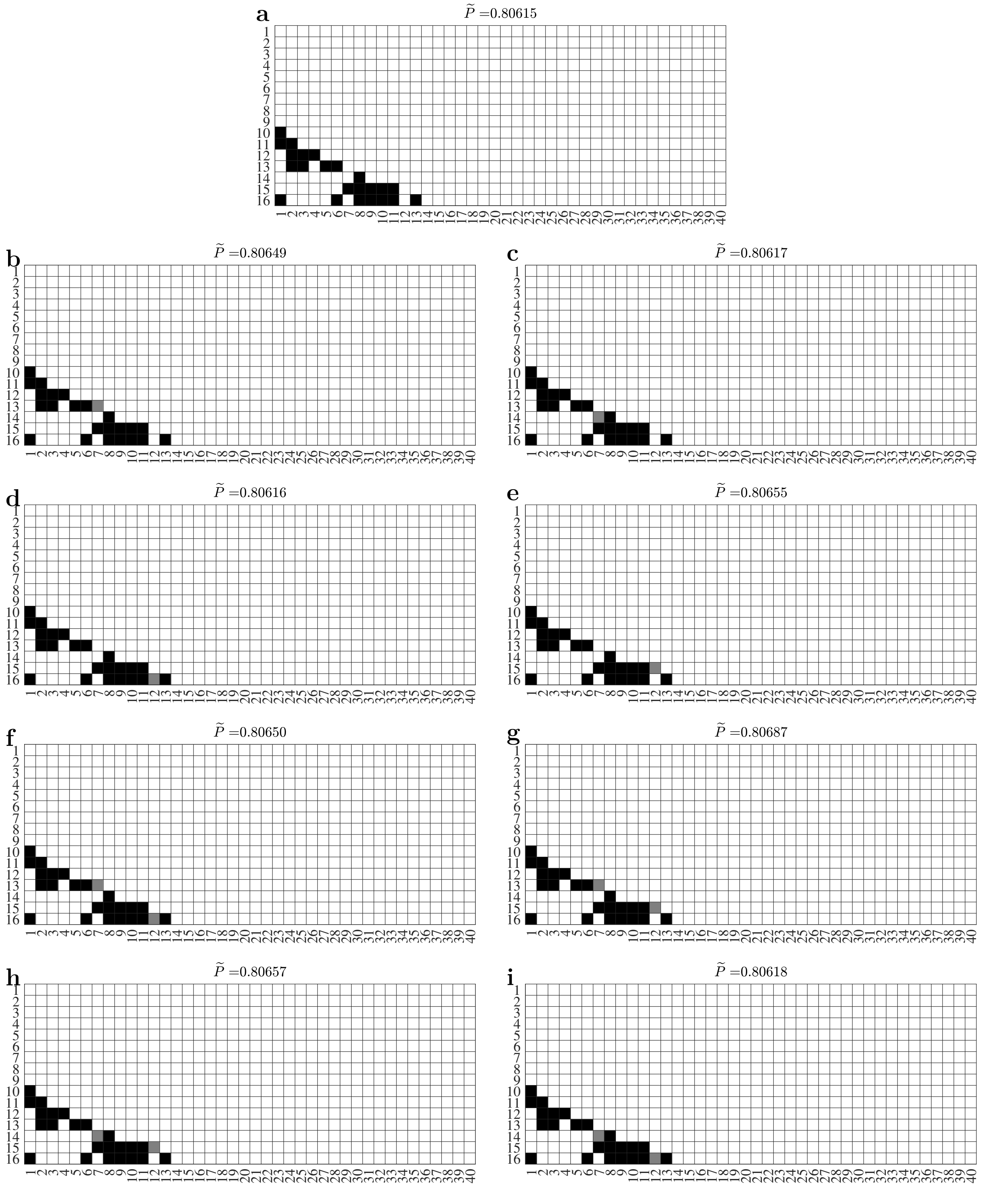

Supplementary Fig. 8: Perturbation of the optimum from SOLO-G for the fluid-structure optimization problem with 40×16 mesh. Intuitively the ramp should be smooth, yet we observe two gaps in the optimum given by SOLO-G. We try filling the gaps. **a**, The optimum from SOLO-G. **b-i**, One or two blocks (gray) are added to fill the gap, with higher $\widetilde{P}$.

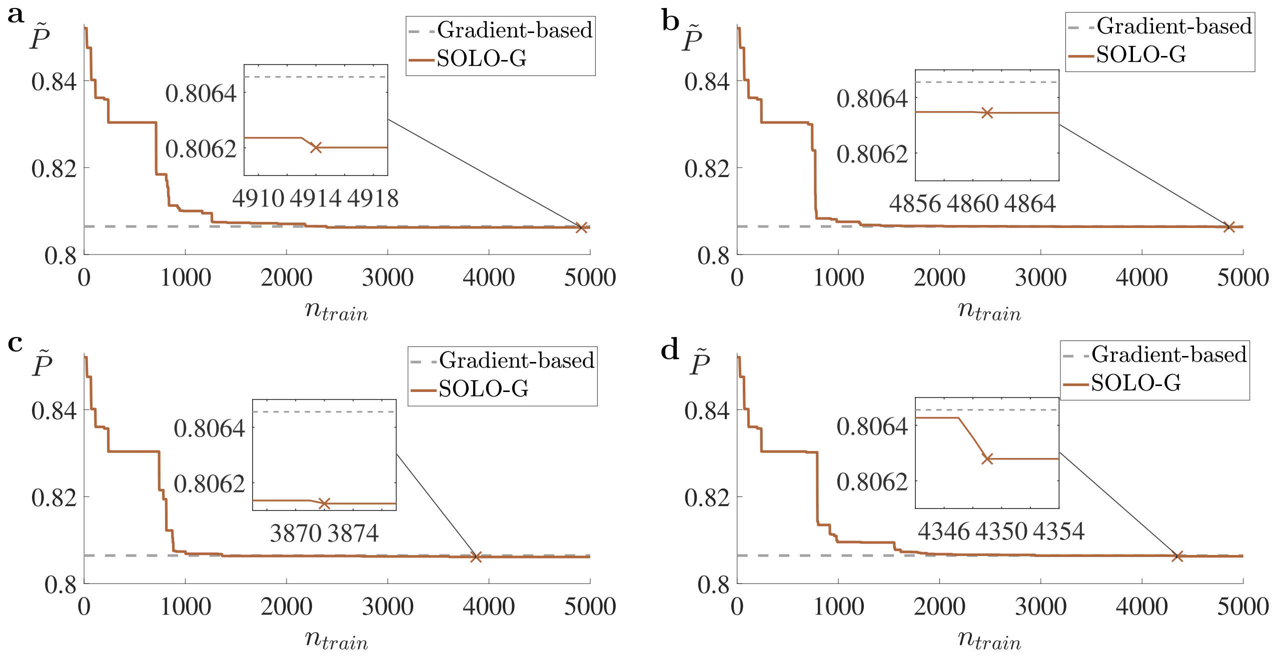

Supplementary Fig. 9: Repeating SOLO-G for the fluid-structure optimization problem with 40×16 mesh. All configurations are the same as Fig. 5b except that different random seeds and higher n_{trian} are used. They all outperform the gradient-based baseline.

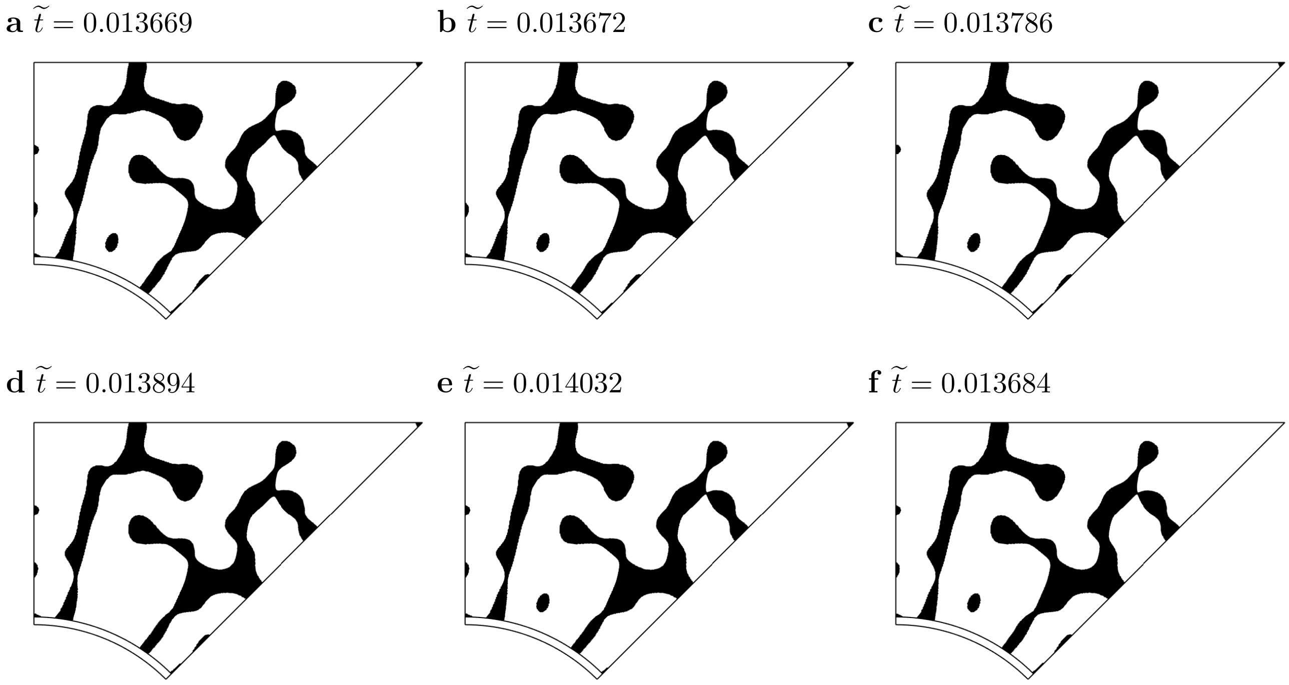

Supplementary Fig. 10: Perturbation of the optimum from SOLO for the heat transfer enhancement problem. **a**, The optimum from SOLO. **b-f**, Copper islands are removed; other copper portions will become thicker to maintain total solid volume. The solution from SOLO gives lowest time $\widetilde{t}$, although some are very close (the difference may even be caused by numerical noise in FEM computation).

2 Theory on convergence

In the main text, we presented a simplified version of convergence (Eq. (3)). In this section, we give a detailed description of our theoretical result. We first present the main result (Theorem 1). Then, we introduce some preliminary definitions and knowledge used in the proof. In the end, we approach the proof.

2.1 Formulation and theorem

The unknown object function is denoted as $F(\boldsymbol{\rho})$, where $\boldsymbol{\rho} \in \mathbb{R}^N$.We denote the domain of $\{\boldsymbol{\rho} \mid 0 \leq \rho_i \leq 1, 1 \leq i \leq N\}$ as K. We suppose the global minimizer $\boldsymbol{\rho}^* = \operatorname{argmin}_{\boldsymbol{\rho}} F(\boldsymbol{\rho})$.

We consider the total iteration number to be T. At iteration $t(1 \leq t \leq T)$, the DNN is denoted as $f_t(\cdot)$ and we denote the empirical minimizer of this DNN function to be $\widehat{\boldsymbol{\rho}}^{(t)}$, i.e.

$$\widehat{\boldsymbol{\rho}}^{(t)} = \underset{\boldsymbol{\rho}}{\operatorname{argmin}}\, f_t(\boldsymbol{\rho}). \tag{S1}$$

Besides, we denote our DNN as a D-layer neural network which is formulated as follows:

$$f_t(\boldsymbol{\rho}) = \mathbf{W}_D^\top \sigma(\mathbf{W}_{D-1}\sigma(...\sigma(\mathbf{W}_1\boldsymbol{\rho}))),$$

where $\mathcal{W} = \{\mathbf{W}_k \in \mathbb{R}^{d_{k-1}\times d_k} \mid k = 1, ..., D\}, d_0 = N$(number of input dimensions), $d_D = 1$, and $\sigma(v) = [\max\{v_1, 0\}, ..., \max\{v_d, 0\}]^\top$ is the ReLU[1] activation function for $v \in \mathbb{R}^d$. We further denote $d = \max\{d_i\}$ and the function class of such neural networks as $\mathcal{H}_f$.

At time step t, given the empirical optimal point $\widehat{\boldsymbol{\rho}}^{(t-1)}$, the additional m training points is generated through the following process:

$$\boldsymbol{\rho}^{(j_t)} = \widehat{\boldsymbol{\rho}}^{(t-1)} + \boldsymbol{\xi}^{(j_t)}, j_t = mt - m + 1, mt - m + 2, \cdots, mt.$$

Here $\boldsymbol{\xi}^{(j)}$ denotes random noise for perturbation. Hence through the iterating process, the sampled points are random variables. At time step t, we denote all the realizations of random training data points set as $K_t = \{\boldsymbol{\rho}^{(i)} \bigm| i = 1, \cdots, mt\}$.

Now before we proceed, we need to impose some mild assumptions on the problem.

Assumption 1. We suppose that

1) the spectral norm of the matrices in DNNs are uniformly bounded, i.e., there exists $B_W > 0$ s.t. $\|\mathbf{W}_k\|_2 \leq B_W, \forall k = 1, \cdots, D$.
2) the target function is bounded, i.e., there exists $B_F > 0$ s.t. $\|F\|_\infty \leq B_F$.

1) of Assumption 1 is a commonly studied assumption in existing generalization theory literature on deep neural networks[2–4]. 2) of Assumption 1 assumes F is bounded, which is standard and intuitive since F has a physical meaning.

Assumption 2. We assume that for any iteration t, $\boldsymbol{\xi}^{(j_t)}$ ($j_t = mt - m + 1, \cdots, mt$) are i.i.d. (independent and identically distributed) perturbation noise. The generated training data $\left\{\boldsymbol{\rho}^{(j_{t_1})}\right\}$ are independent of $\left\{\boldsymbol{\rho}^{(j_{t_2})}\right\}$ if $t_1 \neq t_2$.

The assumption of the i.i.d. properties of noise in Assumption 2 is common in optimization literature[5–8]. The difference is that in traditional optimization literature noise refers to the difference between the true gradient and the stochastic gradient while the noise here denotes perturbations to generate new samples in each iteration. Note that our Assumption 2 only needs the i.i.d. property of noise, which is weaker than the standard assumptions for stochastic gradient methods which require unbiased property and bounded variance[6–8]. Since our fitting DNN f_ts are continuously changing throughout iterations and the empirical minimizers $\widehat{\rho}^{(t)}$ are also alternating, it is reasonable for us to assume that the different groups of generated data samples are independent for the ease of theoretical analysis in the sequel.

We denote the distribution of samples $\{\boldsymbol{\rho}^{(j_t)} \mid j_t = mt - m + 1, mt - m + 2, \cdots, mt\}$ as $D_t (1 \leq t \leq T)$, with which we can introduce the following definition.

Definition 1. For a measurable function f, we denote

$$\mathbb{E}_{D_{1:T}} f(\boldsymbol{\rho}) = \frac{\sum_{t=1}^{T} \mathbb{E}_{\boldsymbol{\rho} \sim D_t} f(\boldsymbol{\rho})}{T}, \tag{S2}$$

where $\mathbb{E}$ denotes expectation.

Assumption 3. For any t and $f_t \in \mathcal{H}_f$,

$$\|F - f_t\|_\infty^2 = C(t) \mathbb{E}_{\boldsymbol{\rho} \sim D_{1:t}} (F - f_t)^2,$$

where $C(t)$ is a monotonically decreasing function w.r.t. iteration number t.

Assumption 3 basically describes that the Chebyshev distance of our DNN at time t and F is bounded by a constant number (w.r.t. t) times the average true loss of $(F - f_t)^2$ till time t. This assumption is reasonable in that the the average true loss can be seen as a variant of Euclidean distance between our DNN at time t and F.

Eventually we arrive at our main result.

Theorem 1. Under Assumptions 1, 2 and 3, given iteration number T and any $\delta > 0$, for any trained DNN $f_T \in \mathcal{H}_f$ with empirical MSE training error ϵ at iteration T, we have that with probability at least $1 - \delta$ over the joint distribution of $\boldsymbol{\rho}^{(1)}, \boldsymbol{\rho}^{(2)}, \cdots, \boldsymbol{\rho}^{(mT)}$,

$$\begin{aligned}
&\left(F(\widehat{\boldsymbol{\rho}}^{(T)}) - F(\boldsymbol{\rho}^*)\right)^2 \\
&\leq 4C(T) \left(\frac{96B^2}{\sqrt{mT}} \sqrt{d^2 D \log\left(1 + 8BB_W^D D\sqrt{mTd}\right)} + 12B^2 \sqrt{\frac{2\log\frac{2}{\delta}}{mT}} + \frac{8}{mT} + \epsilon \right),
\end{aligned}$$

where $B = \max\{B_F, B_W^D\}$.

2.2 Preliminaries

Before showing the complete proof, we introduce some definitions and lemmas.

Lemma 1 (McDiarmid's Inequality[9]). Let $X_1, \cdots, X_m \in \mathcal{X}$ be a set of $m \geq 1$ independent random variables and assume that there exist $c_1, \cdots, c_m > 0$ such that $h : \mathcal{X}^m \to \mathbb{R}$ satisfies the following conditions:

$$|h(x_1, \cdots, x_i, \cdots, x_m) - h(x_1, \cdots, x_i', \cdots, x_m)| \leq c_i,$$

for all $i \in [m]$ and any points $x_1, \cdots, x_m, x_i' \in \mathcal{X}$. Here xs are the realizations of Xs. Let $h(S)$ denote $h(X_1, \cdots, X_m)$, then, for all $s > 0$, the following inequality hold:

$$\mathbb{P}\left\{h(S) - \mathbb{E}[h(S)] \geq s\right\} \leq \exp\left(\frac{-2s^2}{\sum_{i=1}^m c_i^2}\right), \tag{S3}$$

$$\mathbb{P}\left\{h(S) - \mathbb{E}[h(S)] \leq -s\right\} \leq \exp\left(\frac{-2s^2}{\sum_{i=1}^m c_i^2}\right), \tag{S4}$$

where $\mathbb{P}$ denotes probability and $\mathbb{E}$ denotes expectation.

Definition 2 (Covering Number[10]). Let $(V, \|\cdot\|)$ be a normed space, and $\Theta \subset V$. Vector set $\{V_i \in V | i = 1, \cdots, N\}$ is an ι-covering of Θ if $\Theta \subset \cup_{i=1}^N B(V_i, \iota)$ where $B(V_i, \iota)$ denotes the ball with center V_i and radius ι, equivalently, $\forall \theta \in \Theta, \exists i$ such that $\|\theta - V_i\| \leq \iota$. The covering number is defined as :

$$\mathcal{N}(\Theta, \|\cdot\|, \iota) := \min\left\{n : \exists\iota\text{-covering over } \Theta \text{ of size } n\right\}.$$

Definition 3 (Rademacher Complexity & Empirical Rademacher Complexity[10,11]). Given a sample $S = \{\mathbf{x}^{(1)}, \mathbf{x}^{(2)}, \cdots, \mathbf{x}^{(n)}\}$ and a set of real-valued function $\mathcal{H}$, the *Empirical Rademacher Complexity* is defined as

$$\widehat{\mathfrak{R}}_n(\mathcal{H}) = \mathfrak{R}_n(\mathcal{H}_{|S}) := \frac{1}{n}\mathbb{E}_\sigma \sup_{h\in\mathcal{H}} \sum_{i=1}^n \sigma_i h(\mathbf{x}^{(i)}),$$

where sup denotes supremum and the expectation is over the Rademacher random variables $(\sigma_1, \sigma_2, \cdots, \sigma_i, \cdots, \sigma_n)$, which are i.i.d. (independent and identically distributed) with $\mathbb{P}(\sigma_i = 1) = \mathbb{P}(\sigma_i = -1) = \frac{1}{2}$. The *Rademacher Complexity* is defined as

$$\mathfrak{R}_n(\mathcal{H}) := \mathbb{E}_S \mathfrak{R}_n(\mathcal{H}_{|S}) = \frac{1}{n}\mathbb{E}_{S,\sigma} \sup_{h\in\mathcal{H}} \sum_{i=1}^n \sigma_i h(\mathbf{x}^{(i)}),$$

which is the expectation of the Empirical Rademacher Complexity over sample S.

Lemma 2 (Dudley's Entropy Integral Bound[4]). Given a sample $S = \{\mathbf{x}^{(1)}, \mathbf{x}^{(2)}, \cdots, \mathbf{x}^{(n)}\}$, let $\mathcal{H}$ be a real-valued function class taking values in $[0, r]$ for some constant r, and assume that zero function $\mathbf{0} \in \mathcal{H}$. Then we have

$$\widehat{\mathfrak{R}}_n(\mathcal{H}) \leq \inf_{\alpha>0}\left(\frac{4\alpha}{\sqrt{n}} + \frac{12}{n}\int_\alpha^{r\sqrt{n}} \sqrt{\log\mathcal{N}\left(\mathcal{H}, \iota, \|\cdot\|_\infty\right)}d\iota\right),$$

where inf denotes infimum.

Lemma 3. (Covering number bound using volume ratio[4]) Let $\mathcal{W} = \{W \in \mathbb{R}^{a\times b} : \|W\|_2 \leq \lambda\}$ be the set of matrices with bounded spectral norm and ι be given. The covering number $\mathcal{N}(\mathcal{W}, \iota, \|\cdot\|_F)$ is upper bounded by

$$\mathcal{N}\left(\mathcal{W}, \iota, \|\cdot\|_{\mathrm{F}}\right) \leq \left(1 + 2 \cdot \frac{\min\left\{\sqrt{a}, \sqrt{b}\right\}\lambda}{\iota}\right)^{ab}.$$

2.3 Proof

This subsection presents the complete proof of Theorem 1. We first give a proof sketch.

Proof sketch We provide a sketch of proof of Theorem 1 for readers' convenience. First by the property of our algorithm and telescoping, we can get

$$\sup_{f_T\in H_f}\left(F\left(\widehat{\boldsymbol{\rho}}^{(T)}\right) - F\left(\boldsymbol{\rho}^*\right)\right)^2 \leq 4\sup_{f_T\in H_f}\|F - f_T\|_\infty^2. \tag{S5}$$

(S5) means that when function f_T can fit the target function F very well, the universal convergence can be guaranteed. By Assumption 3, we can rewrite (S5) as

$$\sup_{f_T\in H_f}\left(F\left(\widehat{\boldsymbol{\rho}}^{(T)}\right) - F\left(\boldsymbol{\rho}^*\right)\right)^2 \leq 4C(T)\sup_{f_T\in H_f}\frac{\sum_{t=1}^T \mathbb{E}_{\boldsymbol{\rho}\sim D_t}(F(\boldsymbol{\rho}) - f_T(\boldsymbol{\rho}))^2}{T}. \tag{S6}$$

Then we can employ the standard argument of Rademacher Complexity to bound the RHS of (S6) and then obtain

$$\begin{aligned}&\sup_{f_T\in H_f}\frac{\sum_{t=1}^T \mathbb{E}_{\boldsymbol{\rho}\sim D_t}(F(\boldsymbol{\rho}) - f_T(\boldsymbol{\rho}))^2}{T}\\ &\qquad\leq 2\widehat{\mathfrak{R}}_{K_T}(\mathcal{H}_M) + 12B^2\sqrt{\frac{2\log\frac{2}{\delta}}{mT}} + \sup_{f_T\in H_f}\frac{1}{mT}\sum_{i=1}^{mT}\left(F\left(\boldsymbol{\rho}^{(i)}\right) - f_T\left(\boldsymbol{\rho}^{(i)}\right)\right),\end{aligned} \tag{S7}$$

where function class $\mathcal{H}_M = \{(f_T(\boldsymbol{\rho}) - F(\boldsymbol{\rho}))^2 \mid f_T \in \mathcal{H}_f\}$ and $\sup_{f_T\in H_f}\frac{1}{mT}\sum_{i=1}^{mT}\left(F\left(\boldsymbol{\rho}^{(i)}\right) - f_T\left(\boldsymbol{\rho}^{(i)}\right)\right)$ can be viewed as the supreme of the training error (ϵ by our assumption, can be arbitrarily small). Then utilizing Lemma 2, we have

$$\widehat{\mathfrak{R}}_{K_T}(\mathcal{H}_M) \leq \frac{4\alpha}{\sqrt{mT}} + \frac{48B^2}{\sqrt{mT}}\sqrt{\log\mathcal{N}\left(\mathcal{H}_M, \alpha, \|\cdot\|_\infty\right)}, \tag{S8}$$

where $\mathcal{N}$ denotes the covering number. Then through investigating the Lipschitz property of f_T w.r.t to its parameter set, employing the argument of volume ratio (Lemma 3) and setting α as $\frac{1}{\sqrt{mT}}$, we can bound the covering number by

$$\mathcal{N}\left(\mathcal{H}_M, \frac{1}{\sqrt{mT}}, \|\cdot\|_\infty\right) \leq d^2 D\log\left(1 + 8BDB_W^D\sqrt{mTd}\right). \tag{S9}$$

Finally combining (S6), (S7), (S8) and (S9), we get the desired universal convergence result.

Before showing the full proof, we introduce an auxiliary lemma here.

Lemma 4. Under Assumptions 2, we have

1) the whole generated data points $\left\{\boldsymbol{\rho}^{(i)} \mid i = 1, 2, \cdots, mT\right\}$ are mutually independent.
2) for any t, $\left\{\boldsymbol{\rho}^{(j_t)} \mid j_t = mt - m + 1, \cdots, mt\right\}$ are i.i.d..

Lemma 4 is a straightforward result of Assumption 2.

Now we can approach the proof of Theorem 1.

Proof. We first bound term $\sup_{f_T \in H_f}(F(\widehat{\boldsymbol{\rho}}^{(T)}) - F(\boldsymbol{\rho}^*))^2$ by telescoping:

$$\begin{aligned}
&\sup_{f_T \in H_f} (F(\widehat{\boldsymbol{\rho}}^{(T)}) - F(\boldsymbol{\rho}^*))^2 \\
&\quad \overset{\text{(i)}}{\leq} \sup_{f_T \in H_f} (F(\widehat{\boldsymbol{\rho}}^{(T)}) - f_T(\widehat{\boldsymbol{\rho}}^{(T)}) + f_T(\boldsymbol{\rho}^*) - F(\boldsymbol{\rho}^*))^2 \\
&\quad \overset{\text{(ii)}}{\leq} \sup_{f_T \in H_f} 2\{[F(\widehat{\boldsymbol{\rho}}^{(T)}) - f_T(\widehat{\boldsymbol{\rho}}^{(T)})]^2 + [f_T(\boldsymbol{\rho}^*) - F(\boldsymbol{\rho}^*)]^2\} \\
&\quad \leq 4 \sup_{f_T \in H_f} \|F - f_T\|_\infty^2 \\
&\quad \overset{\text{(iii)}}{=} 4C(T) \sup_{f_T \in H_f} \frac{\sum_{t=1}^T \mathbb{E}_{\boldsymbol{\rho} \sim D_t}(F(\boldsymbol{\rho}) - f_T(\boldsymbol{\rho}))^2}{T}.
\end{aligned} \tag{S10}$$

Here (i) comes from Eq. (S1), (ii) uses the fact that for any real number x and y, we have $(x+y)^2 \leq 2(x^2 + y^2)$. (iii) arises from Assumption 3.

For notational simplicity we further denote

$$\Phi(K_T) = \sup_{f_T \in \mathcal{H}_f} \left[\mathbb{E}_{D_{1:T}}(F - f_T)^2 - \widehat{\mathbb{E}}_{K_T}(F - f_T)^2\right], \tag{S11}$$

where $\widehat{\mathbb{E}}_{K_T}(F - f_T)^2 = \frac{1}{mT}\sum_{i=1}^{mT}(F(\boldsymbol{\rho}^{(i)}) - f_T(\boldsymbol{\rho}^{(i)}))$ corresponds to the empirical MSE loss when training our neural network.

Suppose K_T' and K_T are two samples which are different only in the k-th point, namely $K_T = \{\boldsymbol{\rho}^{(1)}, ..., \boldsymbol{\rho}^{(k)}, ..., \boldsymbol{\rho}^{(mT)}\}$ and $K_T' = \{\boldsymbol{\rho}^{(1)}, ..., \boldsymbol{\rho}^{(k)\prime}, ..., \boldsymbol{\rho}^{(mT)}\}$, we have

$$\begin{aligned}
|\Phi(K_T') - \Phi(K_T)| &\leq \sup_{f_T \in \mathcal{H}_f} \left|\widehat{\mathbb{E}}_{K_T}(F - f_T)^2 - \widehat{\mathbb{E}}_{K_T'}(F - f_T)^2\right| \\
&= \sup_{f_T \in \mathcal{H}_f} \left|\frac{(F(\boldsymbol{\rho}^{(k)}) - f_T(\boldsymbol{\rho}^{(k)}))^2}{mT} - \frac{(F(\boldsymbol{\rho}^{(k)\prime}) - f_T(\boldsymbol{\rho}^{(k)\prime}))^2}{mT}\right| \\
&\leq \frac{8B^2}{mT},
\end{aligned}$$

then by Mcdiarmid's Inequality (Eq.(S3) in Lemma 1), we get

$$\begin{aligned}\mathbb{P}(\Phi(K_T) - \mathbb{E}_{K_T}\Phi(K_T) \geq s) &\leq \exp\left(\frac{-2s^2}{mT \cdot (\frac{8B^2}{mT})^2}\right) \\ &= \exp\left(\frac{-mTs^2}{32B^4}\right). \end{aligned} \tag{S12}$$

Given any $\delta > 0$, by setting the right hand side of (S12) to be $\frac{\delta}{2}$, we have with probability at least $1 - \frac{\delta}{2}$,

$$\Phi(K_T) \leq \mathbb{E}_{K_T}\Phi(K_T) + 4B^2\sqrt{\frac{2\log\frac{2}{\delta}}{mT}}. \tag{S13}$$

Notice that

$$\begin{aligned}\mathbb{E}_{K_T}\Phi(K_T) &= \mathbb{E}_{K_T}\left\{\sup_{f_T\in\mathcal{H}_f}\left[\mathbb{E}_{D_{1:T}}(F-f_T)^2 - \widehat{\mathbb{E}}_{K_T}(F-f_T)^2\right]\right\} \\ &= \mathbb{E}_{K_T}\left\{\sup_{f_T\in\mathcal{H}_f}\mathbb{E}_{K'_T}\left[\widehat{\mathbb{E}}_{K'_T}(F-f_T)^2 - \widehat{\mathbb{E}}_{K_T}(F-f_T)^2\right]\right\}. \end{aligned} \tag{S14}$$

Here the second equality in Eq. (S14) is because:

$$\begin{aligned}\mathbb{E}_{K'_T}\left[\widehat{\mathbb{E}}_{K'_T}(F-f_T)^2\right] =& \frac{1}{mT}\sum_{i=1}^{mT}\mathbb{E}_{K'_T}\left[F\left(\boldsymbol{\rho}^{(i)}\right) - f_T(\boldsymbol{\rho}^{(i)})\right]^2 \\ \overset{\text{(i)}}{=}& \frac{1}{mT}\left\{\sum_{i=1}^{m}\mathbb{E}_{\boldsymbol{\rho}^{(i)}\sim D_1}\left[F(\boldsymbol{\rho}^{(i)}) - f_T(\boldsymbol{\rho}^{(i)})\right]^2\right. \\ &+\sum_{i=m+1}^{2m}\mathbb{E}_{\boldsymbol{\rho}^{(i)}\sim D_2}\left[F(\boldsymbol{\rho}^{(i)}) - f_T(\boldsymbol{\rho}^{(i)})\right]^2 + \cdots \\ &\left.+\sum_{i=mT-T+1}^{mT}\mathbb{E}_{\boldsymbol{\rho}^{(i)}\sim D_T}\left[F(\boldsymbol{\rho}^{(i)}) - f_T(\boldsymbol{\rho}^{(i)})\right]^2\right\} \\ \overset{\text{(ii)}}{=}& \frac{1}{mT}\left[m\mathbb{E}_{D_1}(F-f_T)^2 + m\mathbb{E}_{D_2}(F-f_T)^2 + \cdots + m\mathbb{E}_{D_T}(F-f_T)^2\right] \\ =& \mathbb{E}_{D_{1:T}}(F-f_T)^2, \end{aligned}$$

where (i) results from 1) of Lemma 4 and (ii) comes from 2) of Lemma 4.

Further we have

$$
\begin{aligned}
&\mathbb{E}_{K_T}\left\{\sup_{f_T\in\mathcal{H}_f}\mathbb{E}_{K_T'}\left[\widehat{\mathbb{E}}_{K_T'}(F-f_T)^2-\widehat{\mathbb{E}}_{K_T}(F-f_T)^2\right]\right\}\\
\overset{\text{(i)}}{\le}&\mathbb{E}_{K_T,K_T'}\sup_{f_T\in\mathcal{H}_f}\left[\widehat{\mathbb{E}}_{K_T'}(F-f_T)^2-\widehat{\mathbb{E}}_{K_T}(F-f_T)^2\right]\\
=&\mathbb{E}_{K_T,K_T'}\sup_{f_T\in\mathcal{H}_f}\frac{1}{mT}\sum_{i=1}^{mT}\left[(F(\boldsymbol{\rho}^{(i)\prime})-f_T(\boldsymbol{\rho}^{(i)\prime}))^2-(F(\boldsymbol{\rho}^{(i)})-f_T(\boldsymbol{\rho}^{(i)}))^2\right]\\
\overset{\text{(ii)}}{=}&\mathbb{E}_{\sigma,K_T,K_T'}\sup_{f_T\in\mathcal{H}_f}\frac{1}{mT}\sum_{i=1}^{mT}\sigma_i\left[(F(\boldsymbol{\rho}^{(i)\prime})-f_T(\boldsymbol{\rho}^{(i)\prime}))^2-(F(\boldsymbol{\rho}^{(i)})-f_T(\boldsymbol{\rho}^{(i)}))^2\right]\\
\overset{\text{(iii)}}{\le}&\mathbb{E}_{\sigma,K_T'}\sup_{f_T\in\mathcal{H}_f}\frac{1}{mT}\sum_{i=1}^{mT}\left[\sigma_i(F(\boldsymbol{\rho}^{(i)\prime})-f_T(\boldsymbol{\rho}^{(i)\prime}))^2\right]+\mathbb{E}_{\sigma,K_T}\sup_{f_T\in\mathcal{H}_f}\frac{1}{mT}\sum_{i=1}^{mT}\left[-\sigma_i(F(\boldsymbol{\rho}^{(i)})-f_T(\boldsymbol{\rho}^{(i)}))^2\right]\\
=&2\mathbb{E}_{\sigma,K_T}\sup_{f_T\in\mathcal{H}_f}\frac{1}{mT}\sum_{i=1}^{mT}\left[\sigma_i(F(\boldsymbol{\rho}^{(i)})-f_T(\boldsymbol{\rho}^{(i)}))^2\right], \qquad \text{(S15)}
\end{aligned}
$$

where σ_i are Rademacher variables (Definition 3), which are uniformly distributed independent random variables taking values in $\{-1,+1\}$. Here (i) and (iii) hold due to the sub-additivity of the supremum function (considering the convexity of supremum function, by Jensen's Inequality, we have for any function h, $\sup\int_x h(x)\le\int_x\sup h(x)$ holds). (ii) combines the definition of Rademacher variable σ_i and the fact that the expectation is taken over both K_T and $K_{T'}$.

For notational simplity, given any function $f_T\in\mathcal{H}_f$, we define the non-negative loss function $M(f_T):\boldsymbol{\rho}\to(f_T(\boldsymbol{\rho})-F(\boldsymbol{\rho}))^2$ and its function class $\mathcal{H}_M=\{M(f_T):f_T\in\mathcal{H}_f\}$.

Then combining (S14) and (S15) we obtain

$$\mathbb{E}_{K_T}\Phi(K_T)\le 2\mathfrak{R}_{mT}(\mathcal{H}_M), \qquad \text{(S16)}$$

where $\mathfrak{R}_{mT}(\mathcal{H}_M)=\mathbb{E}_{\sigma,K_T}\sup_{f_T\in\mathcal{H}_f}\frac{1}{mT}\sum_{i=1}^{mT}\sigma_i(F(\boldsymbol{\rho}^{(i)})-f_T(\boldsymbol{\rho}^{(i)}))^2$ is the Rademacher Complexity (Definition 3) of $\mathcal{H}_M$.

Now, we define the Empirical Rademacher Complexity of $\mathcal{H}_M$ as

$$\widehat{\mathfrak{R}}_{K_T}(\mathcal{H}_M):=\mathbb{E}_\sigma\sup_{f_T\in\mathcal{H}_f}\frac{1}{mT}\sum_{i=1}^{mT}\sigma_i(F(\boldsymbol{\rho}^{(i)})-f_T(\boldsymbol{\rho}^{(i)}))^2.$$

Again, suppose K_T' and K_T are two samples which are different only in the k-th point, namely

$K_T = \{\boldsymbol{\rho}^{(1)}, ..., \boldsymbol{\rho}^{(k)}, ..., \boldsymbol{\rho}^{(mT)}\}$ and $K'_T = \{\boldsymbol{\rho}^{(1)}, ..., \boldsymbol{\rho}^{(k)\prime}, ..., \boldsymbol{\rho}^{(mT)}\}$, we have

$$\begin{aligned}
&|\widehat{\mathfrak{R}}_{K_T}(\mathcal{H}_M) - \widehat{\mathfrak{R}}_{K'_T}(\mathcal{H}_M)| \\
&= \left|\mathbb{E}_\sigma \sup_{f_T \in \mathcal{H}_f} \frac{1}{mT} \sum_{i=1}^{mT} \sigma_i (F(\boldsymbol{\rho}^{(i)}) - f_T(\boldsymbol{\rho}^{(i)}))^2 - \mathbb{E}_\sigma \sup_{f_T \in \mathcal{H}_f} \frac{1}{mT} \sum_{i=1}^{mT} \sigma_i (F(\boldsymbol{\rho}^{(i)\prime}) - f_T(\boldsymbol{\rho}^{(i)\prime}))^2\right| \\
&\leq \sup_{f_T \in \mathcal{H}_f} \left|\frac{(F(\boldsymbol{\rho}^{(k)}) - f_T(\boldsymbol{\rho}^{(k)}))^2}{mT} - \frac{(F(\boldsymbol{\rho}^{(k)\prime}) - f_T(\boldsymbol{\rho}^{(k)\prime}))^2}{mT}\right| \\
&\leq \frac{8B^2}{mT},
\end{aligned}$$

then by Mcdiarmid's Inequality (Eq.(S4) in Lemma 1), we get

$$\begin{aligned}
\mathbb{P}(\widehat{\mathfrak{R}}_{K_T}(\mathcal{H}_M) - \mathfrak{R}_{mT}(\mathcal{H}_M) \leq -s) &\leq \exp\left(\frac{-2s^2}{mT \cdot (\frac{8B^2}{mT})^2}\right) \\
&= \exp\left(\frac{-mTs^2}{32B^4}\right).
\end{aligned} \tag{S17}$$

Given any $\delta > 0$, by setting the right handside of Eq.(S17) to be $\frac{\delta}{2}$, we have with probability at least $1 - \frac{\delta}{2}$,

$$\mathfrak{R}_{mT}(\mathcal{H}_M) \leq \widehat{\mathfrak{R}}_{K_T}(\mathcal{H}_M) + 4B^2 \sqrt{\frac{2 \log \frac{2}{\delta}}{mT}}. \tag{S18}$$

Now combining (S13), (S16) and (S18), we get with probability at least $1 - \delta$,

$$\Phi(K_T) \leq 2\widehat{\mathfrak{R}}_{K_T}(\mathcal{H}_M) + 12B^2 \sqrt{\frac{2 \log \frac{2}{\delta}}{mT}}, \tag{S19}$$

here we use the fact that (S13) and (S18) hold with probability $1 - \frac{\delta}{2}$ respectively and that $(1 - \frac{\delta}{2})^2 > 1 - \delta$.

It is straightforward that $\|M(f_T)\|_\infty \leq 4B^2$, then Dudley's Entropy (Lemma 2) gives us

$$\begin{aligned}
\widehat{\mathfrak{R}}_{K_T}(\mathcal{H}_M) &\leq \frac{4\alpha}{\sqrt{mT}} + \frac{12}{mT} \int_\alpha^{4B^2\sqrt{mT}} \sqrt{\log \mathcal{N}(\mathcal{H}_M, \iota, \|\cdot\|_\infty)} d\iota \\
&\leq \frac{4\alpha}{\sqrt{mT}} + \frac{48B^2}{\sqrt{mT}} \sqrt{\log \mathcal{N}(\mathcal{H}_M, \alpha, \|\cdot\|_\infty)},
\end{aligned} \tag{S20}$$

where $\mathcal{N}$ denotes the covering number. We pick $\alpha = \frac{1}{\sqrt{mT}}$, and combine (S10), (S19) and (S20) to get

$$\begin{aligned}
&\sup_{f_T \in H_f} (F(\widehat{\boldsymbol{\rho}}^{(T)}) - F(\boldsymbol{\rho}^*))^2 \\
&\leq 4C(T) \left(\frac{96B^2}{\sqrt{mT}} \sqrt{\log \mathcal{N}\left(\mathcal{H}_M, \frac{1}{\sqrt{mT}}, \|\cdot\|_\infty\right)} + 12B^2 \sqrt{\frac{2 \log \frac{2}{\delta}}{mT}} + \frac{8}{mT} + \widehat{\mathbb{E}}_{K_T}(F - f_T)^2\right).
\end{aligned} \tag{S21}$$

Next we need to compute the covering number $\mathcal{N}(\mathcal{H}_M, \frac{1}{\sqrt{mT}}, \|\cdot\|_\infty)$. To derive a tight covering number we investigate the Lispchitz continuity of f_T with respect to the weight matrices $\mathbf{W}_1, \cdots, \mathbf{W}_D$. Consider two neural networks $f_T(\boldsymbol{\rho}) = \mathbf{W}_D^\top \sigma(\mathbf{W}_{D-1}\sigma(...\sigma(\mathbf{W}_1\boldsymbol{\rho})))$ and $f'_T(\boldsymbol{\rho}) = \mathbf{W}_D'^\top \sigma(\mathbf{W}'_{D-1}\sigma(...\sigma(\mathbf{W}'_1\boldsymbol{\rho})))$ with different sets of weight matrices, we first notice that

$$\begin{aligned}\|M(f_T) - M(f'_T)\|_\infty &= \sup_{\boldsymbol{\rho}} \left|(f_T(\boldsymbol{\rho}) - F(\boldsymbol{\rho}))^2 - (f'_T(\boldsymbol{\rho}) - F(\boldsymbol{\rho}))^2\right| \\ &= \sup_{\boldsymbol{\rho}} \left|(f_T(\boldsymbol{\rho}) + f'_T(\boldsymbol{\rho}) - 2F(\boldsymbol{\rho}))(f_T(\boldsymbol{\rho}) - f'_T(\boldsymbol{\rho}))\right| \\ &\leq 4B \left\|f_T - f'_T\right\|_\infty.\end{aligned}$$

Next we get the bound based on weight matrices. Specifically, given two different sets of matrices $\mathbf{W}_1, \cdots, \mathbf{W}_D$ and $\mathbf{W}'_1, \cdots, \mathbf{W}'_D$, we have

$$\begin{aligned}&\|f_T - f'_T\|_\infty \\ &\leq \left\|\mathbf{W}_D^\top \sigma(\mathbf{W}_{D-1}\sigma(...\sigma(\mathbf{W}_1\boldsymbol{\rho})...)) - (\mathbf{W}'_D)^\top \sigma(\mathbf{W}'_{D-1}\sigma(...\sigma(\mathbf{W}'_1\boldsymbol{\rho})...))\right\|_2 \\ &\leq \left\|\mathbf{W}_D^\top \sigma(\mathbf{W}_{D-1}\sigma(...\sigma(\mathbf{W}_1\boldsymbol{\rho})...)) - (\mathbf{W}'_D)^\top \sigma(\mathbf{W}_{D-1}\sigma(...\sigma(\mathbf{W}_1\boldsymbol{\rho})...))\right\|_2 \\ &\quad + \left\|(\mathbf{W}'_D)^\top \sigma(\mathbf{W}_{D-1}\sigma(...\sigma(\mathbf{W}_1\boldsymbol{\rho})...)) - (\mathbf{W}'_D)^\top \sigma(\mathbf{W}'_{D-1}\sigma(...\sigma(\mathbf{W}'_1\boldsymbol{\rho})...))\right\|_2 \\ &\leq \left\|\mathbf{W}_D - \mathbf{W}'_D\right\|_2 \left\|\sigma(\mathbf{W}_{D-1}\sigma(...\sigma(\mathbf{W}_1\boldsymbol{\rho})...))\right\|_2 \\ &\quad + \left\|\mathbf{W}'_D\right\|_2 \left\|\sigma(\mathbf{W}_{D-1}\sigma(...\sigma(\mathbf{W}_1\boldsymbol{\rho})...)) - \sigma(\mathbf{W}'_{D-1}\sigma(...\sigma(\mathbf{W}'_1\boldsymbol{\rho})...))\right\|_2.\end{aligned}$$

Note that we have

$$\begin{aligned}\left\|\sigma(\mathbf{W}_{D-1}\sigma(...\sigma(\mathbf{W}_1\boldsymbol{\rho})...))\right\|_2 &\overset{\text{(i)}}{\leq} \left\|\mathbf{W}_{D-1}\sigma(...\sigma(\mathbf{W}_1\boldsymbol{\rho})...)\right\|_2 \\ &\leq \left\|\mathbf{W}_{D-1}\right\|_2 \left\|\sigma(...\sigma(\mathbf{W}_1\boldsymbol{\rho})...)\right\|_2 \overset{\text{(ii)}}{\leq} B_W^{D-1}\left\|\boldsymbol{\rho}\right\|_2 \overset{\text{(iii)}}{\leq} B_W^{D-1},\end{aligned}$$

where (i) comes from the definition of the ReLU activation, (ii) comes from $\|\mathbf{W}_k\|_2 \leq B_W$ and recursion, and (iii) comes from the boundedness of $\boldsymbol{\rho}$. Accordingly, we have

$$\begin{aligned}\|M(f_T) - M(f'_T)\|_\infty &\leq 4B \left\|f_T(\boldsymbol{\rho}) - f'_T(\boldsymbol{\rho})\right\|_\infty \\ &\leq 4B(B_W^{D-1}\left\|\mathbf{W}_D - \mathbf{W}'_D\right\|_2 + \left\|\mathbf{W}'_D\right\|_2 \left\|\sigma(\mathbf{W}_{D-1}\sigma(...)) - \sigma(\mathbf{W}'_{D-1}\sigma(...))\right\|_2) \\ &\overset{\text{(i)}}{\leq} 4B(B_W^{D-1}\left\|\mathbf{W}_D - \mathbf{W}'_D\right\|_2 + B_W\left\|\mathbf{W}_{D-1}\sigma(...) - \mathbf{W}'_{D-1}\sigma(...)\right\|_2) \\ &\overset{\text{(ii)}}{\leq} 4BB_W^{D-1}\sum_{k=1}^{D}\left\|\mathbf{W}_k - \mathbf{W}'_k\right\|_2, \end{aligned} \tag{S22}$$

where (i) comes from the fact that $\forall \mathbf{A}_1, \mathbf{A}_2 \in \mathbb{R}^{a\times b}$, $\|\sigma(\mathbf{A}_1) - \sigma(\mathbf{A}_2)\|_2 \leq \|\mathbf{A}_1 - \mathbf{A}_2\|_2$, and (ii) comes from the recursion. Considering the fact that each $M(f_T)$ corresponds to its parameter set $\mathbf{W}_1, \cdots, \mathbf{W}_D$, we can then derive the covering number of $\mathcal{H}_M$ by the Cartesian product of

the matrix covering of $\mathbf{W}_1, ..., \mathbf{W}_D$:

$$\begin{aligned}\mathcal{N}(\mathcal{H}_M, \|\cdot\|_\infty, \iota) &\overset{\text{(i)}}{\leq} \prod_{k=1}^{D} \mathcal{N}\left(\mathbf{W}_k, \frac{\iota}{4BB_W^{D-1}D}, \|\cdot\|_2\right) \\ &\overset{\text{(ii)}}{\leq} \prod_{k=1}^{D} \mathcal{N}\left(\mathbf{W}_k, \frac{\iota}{4BB_W^{D-1}D}, \|\cdot\|_\mathrm{F}\right) \\ &\overset{\text{(iii)}}{\leq} \left(1 + \frac{8BB_W^D D\sqrt{d}}{\iota}\right)^{d^2 D}. \end{aligned} \tag{S23}$$

Here (i) utilizes the fact that if $\forall k = 1, 2, \cdots, D$, matrix set

$$\left\{\mathbf{V}_{k,j_k} \in \mathbb{R}^{d_{k-1}\times d_k} \;\middle|\; j_k = 1, 2, \cdots, \mathcal{N}\left(\mathbf{W}_k, \frac{\iota}{4BB_W^{D-1}D}, \|\cdot\|_2\right)\right\}$$

is a $\frac{\iota}{4BB_W^{D-1}D}-$covering of set $\{\mathbf{W}_k \mid \|\mathbf{W}_k\|_2 \leq B_W\}$, then by (S22) we have function set

$$\left\{\mathbf{V}_{D,j_D}^\top \sigma(\mathbf{V}_{D-1,j_{D-1}}\sigma(...\sigma(\mathbf{V}_{1,j_1}\boldsymbol{\rho})...)) \;\middle|\; 1 \leq j_k \leq \mathcal{N}\left(\mathbf{W}_k, \frac{\iota}{4BB_W^{D-1}D}, \|\cdot\|_2\right), \forall 1 \leq k \leq D\right\}$$

is an $\iota-$covering of $\mathcal{H}_M$. (ii) comes from the fact that for any matrix W we have $\|W\|_2 \leq \|W\|_\mathrm{F}$, and (iii) employs Lemma 3. Plugging (S23) into (S21), we get

$$\begin{aligned}&\sup_{f_T \in H_f} (F(\widehat{\boldsymbol{\rho}}^{(T)}) - F(\boldsymbol{\rho}^*))^2 \\ &\leq 4C(T)\left(\frac{96B^2}{\sqrt{mT}}\sqrt{d^2 D \log\left(1 + 8BDB_W^D\sqrt{mTd}\right)} + 12B^2\sqrt{\frac{2\log\frac{2}{\delta}}{mT}} + \frac{8}{mT} + \widehat{\mathbb{E}}_{K_T}(F - f_T)^2\right).\end{aligned} \tag{S24}$$

Since we consider the empirical MSE training loss to be less than ϵ, i.e.,

$$\widehat{\mathbb{E}}_{K_T}(F - f_T)^2 \leq \epsilon, \tag{S25}$$

so by plugging (S25) into (S24), we get the desired result. □

Supplementary References

[1] Nair, V. & Hinton, G. E. Rectified linear units improve restricted boltzmann machines. In *ICML* (2010).

[2] Bartlett, P. L., Foster, D. J. & Telgarsky, M. J. Spectrally-normalized margin bounds for neural networks. In *Advances in Neural Information Processing Systems*, 6240–6249 (2017).

[3] Neyshabur, B., Bhojanapalli, S. & Srebro, N. A pac-bayesian approach to spectrally-normalized margin bounds for neural networks. *arXiv preprint arXiv:1707.09564* (2017).

[4] Chen, M., Li, X. & Zhao, T. On generalization bounds of a family of recurrent neural networks. *arXiv preprint arXiv:1910.12947* (2019).

[5] Blair, C. Problem complexity and method efficiency in optimization (as nemirovsky and db yudin). *SIAM Review* **27**, 264 (1985).

[6] Nemirovski, A., Juditsky, A., Lan, G. & Shapiro, A. Robust stochastic approximation approach to stochastic programming. *SIAM Journal on optimization* **19**, 1574–1609 (2009).

[7] Lin, T., Jin, C. & Jordan, M. I. On gradient descent ascent for nonconvex-concave minimax problems. *arXiv preprint arXiv:1906.00331* (2019).

[8] Chen, M. *et al.* On computation and generalization of generative adversarial imitation learning. *arXiv preprint arXiv:2001.02792* (2020).

[9] McDiarmid, C. Concentration. In *Probabilistic methods for algorithmic discrete mathematics*, 195–248 (Springer, 1998).

[10] Mohri, M., Rostamizadeh, A. & Talwalkar, A. *Foundations of machine learning* (MIT press, 2018).

[11] Bartlett, P. L. & Mendelson, S. Rademacher and gaussian complexities: Risk bounds and structural results. *Journal of Machine Learning Research* **3**, 463–482 (2002).
